# Metabolic differences in women with premature ovarian insufficiency: a systematic review and meta-analysis

**DOI:** 10.1186/s13048-022-01041-w

**Published:** 2022-09-30

**Authors:** Wang-Yu Cai, Xi Luo, Wei Wu, Jianyuan Song, Ning-Ning Xie, Cuicui Duan, Xiao-Ke Wu, Jian Xu

**Affiliations:** 1 Fourth Affiliated Hospital, Zhejiang University School of Medicine, No. 1 Shang Cheng Avenue, Yiwu, 322000 Zhejiang China; 2 Department of Obstetrics and Gynecology, The Second Affiliated Hospital of Zhejiang Chinese Medical University, Hangzhou, China; 3 Center of Reproductive Medicine, The First Affiliated Hospital of Zhengzhou University, Zhengzhou, China; 4 Women’s Hospital, Zhejiang University School of Medicine, Hangzhou, China; 5 Department of Obstetrics and Gynecology, First Affiliated Hospital, Heilongjiang University of Chinese Medicine, Harbin, China; 6 Heilongjiang Province Hospital, Harbin, China

**Keywords:** Premature ovarian insufficiency, Metabolic, Lipid, Glucose, Systematic review

## Abstract

**Objective:**

This review aimed to investigate the metabolic profile of women with premature ovarian insufficiency (POI) compared relative to women with normal ovarian functioning.

**Methods:**

A systematic search of PubMed, EMBASE, and the Web of Science for observational studies published up until the 6^th^ of July 2021 that compared the metabolic profile of POI women with a healthy control group were assessed. Mean differences (MD) and 95% confidence interval (CI) were pooled using the fixed or random effect models.

**Results:**

A total of 21 studies involving 1573 women with POI and 1762 control women were included. POI patients presented significantly higher waist circumference, total cholesterol, low-density lipoprotein, high-density lipoprotein, triglycerides, and fasting glucose. Additionally, POI patients had marginally higher insulin level. However, the differences in systolic, and diastolic blood pressure were non-significant relative to the control group.

**Conclusions:**

POI is associated with alterations in certain metabolic parameters compared to control women. This finding highlights the importance of early screening and the lifelong management of metabolic health for women with POI.

**Supplementary Information:**

The online version contains supplementary material available at 10.1186/s13048-022-01041-w.

## Background

Premature ovarian insufficiency (POI) is described as amenorrhea due to loss of ovarian function before the age of 40 [1, 2]. Additionally, it is characterized by abnormally increased levels of gonadotrophins and decreased levels of estrogen [3]. Although the cause of POI is unclear, it is hypothesized that hormonal and metabolic abnormalities, infections, environmental exposures, medical treatments, endocrinology disorders, and autoimmune diseases may all contribute to this condition [4]. Most women with POI develop symptoms of estrogen deficiency, including vasomotor flushes, vaginal dryness, sexual dysfunction, osteoporosis, and long-term cardiovascular disease [5, 6]. POI is also associated with lower health-related quality of life compared to normal ovarian controls. Further, these patients require additional emotional support from clinicians [7, 8].

It has been suggested that natural and surgical menopause are associated with a higher incidence of a composite of cardiovascular disease (CVD) [9]. Previous systematic reviews have also revealed that women with premature or early menopause exhibit an increased risk of developing and dying from ischaemic heart disease and total CVD [10]. Accumulating studies have shown that women with POI may also be at increased risk of cardiovascular disease, and the risk may be explained in part by metabolic and endothelial changes facilitated by estrogen deprivation [6]. However, the underlying mechanism between the elevated risk of CVD and women with POI still needs answers.

Thus far, some case–control studies have reported differences in certain metabolic parameters between women with POI and healthy controls; however, no comprehensive review exists on this topic. Within this context, this review aims to provide comprehensive guidance and assessment practices for POI through a systematic review and meta-analysis of the metabolic profiles of POI patients relative to healthy controls. Further, we also aim to discuss the metabolic functioning and its potential contribution to POI.

## Methods

### Search strategy

This systematic review was constructed according to the Preferred Reporting Items for Systematic Reviews and Meta-analyses (PRISMA) [11] (Supplementary table 1). A protocol was registered on INPLASY (INPLASY2021100091). Using the combination of keywords provided in Supplementary Table 2, major electronic databases including PubMed, Embase, and Web of Science were used to source relevant literature published up until the 6^th^ of July 2021. Key search terms included: “premature ovarian insufficiency”, “metabolic”, and “case–control”. References from all included studies were also assessed to identify relevant articles not captured by the electronic searches.

### Inclusion and exclusion criteria

Observational studies that compared at least one of the metabolic outcomes of interest in patients with POI to control women with normal ovarian function were included. Metabolic parameters included waist circumference (WC), systolic blood pressure (SBP), diastolic blood pressure (DBP), fasting glucose (FG), insulin (INS), total cholesterol (TC), high-density lipoprotein (HDL), low-density lipoprotein (LDL), and triglycerides (TG). Review articles, opinions, book chapters, letters, published abstracts, animal studies, case reports and studies with no suitable control group were excluded. Only articles with English language were included.

### Study selection

Two authors (WYC and XL) independently scrutinized the titles and abstracts of all studies to identify relevant studies according to the inclusion and exclusion criteria. Full manuscripts of the relevant studies considered for inclusion were then carefully reviewed to include eligible studies. Any disagreement between the two authors was resolved by a third author (JX).

### Data extraction

Two authors (WYC and XL) independently extracted data using the following form: the first author, year of publication, geographic region, sample size, study design, age of case and control, body mass index (BMI) of case and control, follicle-stimulating hormone (FSH) level, estradiol (E2) level, outcome measures and confounding factors controlled for (including but not limited to hormone therapy) were recorded. Where a study with two or more publications was identified, only the most comprehensive or the most recent version was included. For continuous measures, mean and standard deviation was first recorded, for publications that only reported median and interquartile range, the mean and standard deviation was estimated [12].

### Quality assessment

The quality of eligible observational studies was assessed using the Newcastle–Ottawa scale (NOS) [13]. The NOS assesses studies by scoring three aspects: viz selection, comparability, and exposure, The NOS total is scored out of 9 (the higher the score, the better). Each article was awarded a score out of four for selection bias (adequate definition of case, representativeness of the case, selection of control, definition of control), two for comparability (comparability between case and control), and four for bias in the exposure (ascertainment of exposure, consistency of the method of ascertainment for case and control, and non-response rate). The quality of studies was defined as high with NOS scores > 6, medium 4–6, and low < 4.

### Statistical analyses

Review Manager version 5.4.1 and Stata version 8.0 were used to analyze the extracted data. Mean difference (MD) with 95% confidence interval (CI) was pooled to measure effect size. The heterogeneity of studies was measured using the I^2^ index: below 40% indicated no heterogeneity; more than 40% indicated heterogeneity existed. The fixed-effects model was used when no heterogeneity was observed, and the random-effects model was used when heterogeneity existed. Publication bias was assessed using funnel plot asymmetry and Egger’s line regression test. To measure the effect of confounders on the effect size of potential moderators, subgroup analysis and meta-regression were performed. To confirm the robustness of the results, sensitivity analysis was performed by excluding each one included study. A P-value less than 0.05 was considered statistically significant.

## Results

**Fig. 1:**
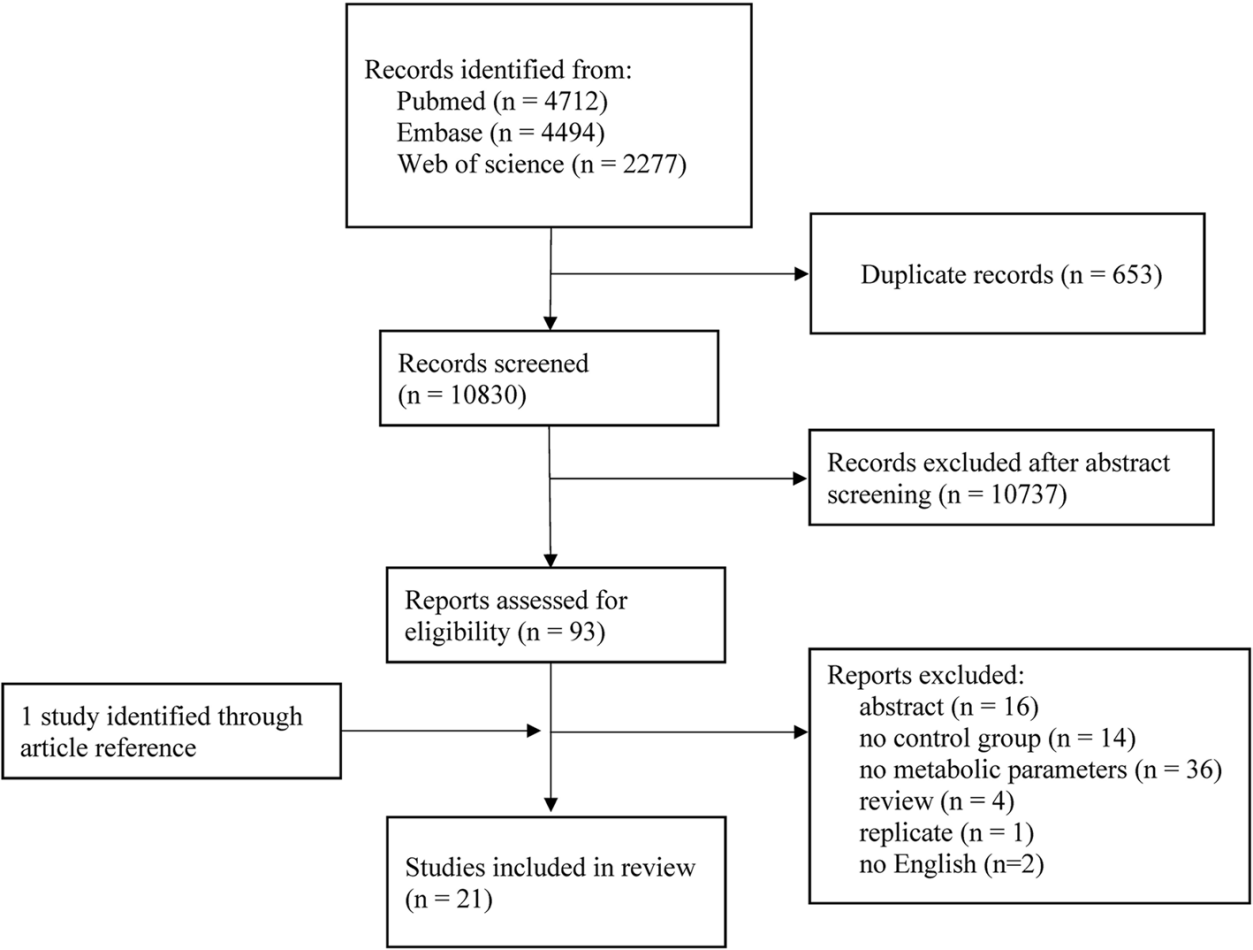
Flowchart for selecting studies

According to the inclusion and exclusion criteria, a total of 21 studies including 1573 women with POI and 1762 control women were utilized in this review (Fig. 1). Following title and abstract screening of the literature search results, 11,483 total studies were assessed of which 653 were duplicates and 10,737 were considered irrelevant. Of the remaining 93 records, 73 records were excluded due to abstract (*n* = 16), no control group (*n* = 14), no metabolic parameters (*n* = 36), the presence of review articles (*n* = 4), replicates (*n* = 1), not in the English language (*n* = 2) (Fig. 1). An additional study was identified through the assessment of the article references. Therefore, a total of 21 studies were eligible for data extraction and were included in the present meta-analysis [14–34].

### Characteristics of included studies

The characteristics of the reviewed studies are presented in Table 1. Among the studies assessed, 8 were conducted in the Middle East, 8 in Europe, 3 in East Asia, one in Africa, and one in Latin America. All studies had a case–control design. The participant’s mean age ranged from 26.4 to 49.9 years. Thirteen studies diagnosed POI by a cutoff of 40 IU/L for FSH [15–17, 19–22, 24, 25, 28, 29, 31, 32], four studies used a cutoff of 25 IU/L [23, 27, 30, 33] and 4 studies didn’t report [14, 18, 26, 34]. Nine studies reported that the POI women had normal chromosomal constitutions [16, 21, 23, 24, 27, 30–33]. WC, SBP and DBP were assessed in 5 [16, 20, 21, 25, 28], 6 [16, 17, 20, 24, 29, 34], and 6 [16, 17, 20, 24, 29, 34] of the studies, respectively. TC, HDL, LDL, TG, FG and INS were measured in 17 [14–26, 29, 32–34], 14 [14, 16–26, 29, 34], 14 [14, 16–26, 29, 34], 15 [14–19, 21–26, 29, 32, 34], 14 [15–19, 21, 23, 24, 26, 29–32, 34], and 7 [16, 19, 26, 27, 29–31] of the studies, respectively. Quality assessment data of the reviewed studies are presented in Table 2, and all included studies had medium to high quality.

**Table 1:** Characteristics of included studies

Author/year published	Country	POI					Control			Outcome measurements	Matched variables
		**Number of** **cases**	**Age** **of cases**	**BMI**	**FSH**	**E2**	**Number of** **controls**	**Age**	**BMI**		
AbdulAzeez 2018 [14]	Nigeria	50	26.4 ± 5.2	Not reported	23.0 ± 15.0	101.8 ± 42.3	40	26.4 ± 5.4	Not reported	TC, TG, HDL, LDL	Age
Ağaçayak 2016 [15]	Turkey	30	28.9 ± 6.8	24.1 ± 4.2	60 ± 20	17.0 ± 7.2	30	29.2 ± 5.0	23.2 ± 3.3	FG, TC, TG	Age, ethnic
Ates 2014 [16]	Turkey	56	35.23 ± 4.58	25.79 ± 4.10	75.33 ± 39.73	47.44 ± 49.48	59	35.47 ± 4.49	26.09 ± 3.80	WC, SBP, DBP, TC, TG, HDL, LDL, FG, INS	Age
Bozkaya 2020 [17]	Turkey	34	30.94 ± 5.58	25.90 ± 4.22	56.68 ± 28.84	32.32 ± 34.70	35	28.53 ± 5.09	24.47 ± 3.89	SBP, DBP, FG, TC, TG, HDL, LDL	
Cekici 2021 [18]	Turkey	66	32.5 ± 4.7	23.6 ± 4.3	75.1 ± 34.9	5.4 ± 1.4	73	31.0 ± 4.9	22.7 ± 5.3	FG, TC, TG, HDL, LDL	Age
Czyzyk 2017 [19]	Poland	23	31 ± 7	24.37 ± 4.35	95.29 ± 48.36	29.26 ± 31.29	18	31 ± 3	24.44 ± 3.18	INS, FG, TC, TG, HDL, LDL	Age, BMI
Daan 2017 [20]	Netherland	83	49.9 ± 4.7	25.1 ± 4.1	NA	5.01 [358.49]	267	50.9 ± 3.1	26.9 ± 4.9	WC, SBP, DBP, TC, HDL, LDL	
Goldmeier 2014 [21]	Brazil	17	37 ± 9	26 ± 5	53.9 ± 24.15	17.7 ± 14.9	15	38 ± 9	26 ± 3	WC, FG, TG, LDL, HDL, TC	Age
Gulhan 2012 [22]	Turkey	47	36.8 ± 1.8	25.9 ± 4.9	94.9 ± 36.4	27.9 ± 28.3	60	36.0 ± 2.3	24.8 ± 4.6	TC, TG, HDL, LDL	Age
Huang 2021 [23]	China	303	34.78 ± 5.42	21.44 ± 2.75	73.23 (51.19 92.85)	7.64 (5.00 28.10)	303	34.68 ± 4.80	21.25 ± 2.92	TC, TG, HDL, LDL, FG	Age
Kalantaridou 2004 [24]	Greece	18	35.4 ± 5.5	24.2 ± 3.1	78.9 ± 22.9	37 ± 24	20	35.0 ± 3.6	23.0 ± 2.6	SBP, DBP, FG, TC, TG, HDL, LDL	Age, BMI
Knauff 2008 [25]	Netherland	90	33.8 ± 5.6	24.9 ± 4.3	79.5 ± 42.3	38.14 ± 41.41	198	30.3 ± 2.9	24.3 ± 4.8	WC, TC, TG, HDL, LDL	
Kulaksizoglu 2013 [26]	Turkey	43	36.83 ± 2.72	30.33 ± 5.59	48.55 ± 16.63	12.62 ± 9.13	33	37.15 ± 1.88	29.62 ± 3.48	FG, INS, TC, TG, HDL, LDL	
Kunicki 2018 [27]	Poland	98	31.48 ± 6.07	23.78 ± 3.563	69.71 ± 25.43	22.34 ± 31.69	78	27.78 ± 5.25	22.20 ± 2.94	INS	
Luo 2018 [28]	China	240	31.58 ± 6.83	21.11 ± 2.57	92.24 ± 33.76	20.46 ± 9.97	240	31.32 ± 6.62	20.90 ± 2.38	WC	Age, BMI
Podfigurna 2018 [29]	Poland	56	30.7 ± 6.9	23.54 ± 4.55	97.57 ± 42.30	21.45 ± 43.07	68	27.3 ± 4.5	28.62 ± 5.30	SBP, DBP, FG, INS, TC, TG, HDL, LDL	Age, weight
Podfigurna 2020 [30]	Poland	132	31.86 ± 7.75	23.68 ± 4.42	100.14 ± 36.93	13.43 ± 18.91	17	23.21 ± 5.86	20.71 ± 5.15	FG, INS	Age, weight
Szlendak-Sauer 2016 [31]	Poland	98	30 ± 6.3	23.5 ± 2.8	73.9 ± 26.8	14.5 ± 8.4	75	29.4 ± 4	23.2 ± 2.5	INS, FG	
Tunc 2017 [32]	Turkey	30	28.9 ± 6.8	25.9 ± 4.1	60.09 ± 20.01	33.75 ± 39.70	30	29.2 ± 5.0	24.1 ± 4.2	TC, TG, FG	Age, gravidity, BMI
Xu 2019 [33]	China	33	Not reported	Not reported	59.56 ± 32.67	Not reported	72	Not reported	Not reported	TC	
Yorgun 2012 [34]	Turkey	26	37.5 ± 10.1	26.0 ± 3.6	47.9 ± 17.5	25.0 ± 15.6	31	37.5 ± 9.0	23.3 ± 1.3	SBP, DBP, TC, TG, LDL, HDL, FG	Age

The data are presented as mean ± standard deviation, median (quartile1, quartile 3) and median [range]

*BMI* Body mass index, *FSH* Follicle-stimulating hormone, *E2* Estradiol, *WC* Waist circumference, *FG* Fasting blood glucose, *INS* Insulin, *SBP* Systolic blood pressure, *DBP* Diastolic blood pressure, *TC* Total cholesterol, *HDL* High-density lipoprotein, *LDL* Low-density lipoprotein and *TG* Triglycerides

**Table 2:** Quality of included studies

	**Selection**				**Comparability**		**Exposure**			
**Author/year** **published**	**Adequate Case Definition**	**Representative** **of cases**	**Selection of controls**	**Definition of Controls**	**Age**	**Other factors**	**Ascertainment of exposure**	**Same** **ascertainment**	**Non-Response Rate**	**Total**
AbdulAzeez 2018 [14]					*		*	*	*	4
Ağaçayak 2016 [15]	*				*	*	*	*	*	6
Ates 2014 [16]	*	*		*	*		*	*	*	7
Bozkaya 2020 [17]	*			*			*	*	*	5
Cekici 2021 [18]		*		*	*		*	*	*	6
Czyzyk 2017 [19]	*			*	*	*	*	*	*	7
Daan 2017 [20]	*			*			*	*	*	5
Goldmeier 2014 [21]	*			*	*		*	*	*	6
Gulhan 2012 [22]	*			*	*		*	*	*	6
Huang 2021 [23]	*			*	*		*	*	*	6
Kalantaridou 2004 [24]	*			*	*	*	*	*	*	7
Knauff 2008 [25]	*	*					*	*	*	5
Kulaksizoglu 2013 [26]	*			*			*	*	*	5
Kunicki 2018 [27]	*			*			*	*	*	5
Luo 2018 [28]	*				*	*	*	*	*	6
Podfigurna 2020 [29]	*			*	*	*	*	*	*	7
Podfigurna 2018 [30]	*			*	*	*	*	*	*	7
Szlendak-Sauer 2016 [31]	*			*			*	*	*	5
Tunc 2017 [32]	*			*	*	*	*	*	*	7
Xu 2019 [33]	*			*			*	*	*	5
Yorgun 2012 [34]	*	*			*		*	*	*	6

### Waist circumference

**Fig. 2:**
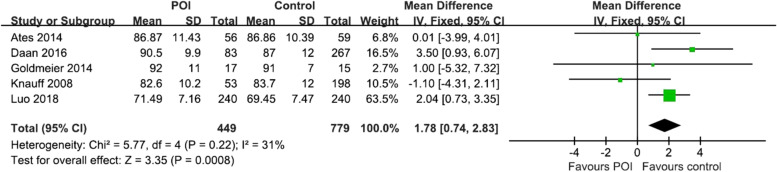
Forest plot for the meta-analysis for waist circumference between premature ovarian insufficiency and control group

WC was measured in 5 of the studies which included 449 POI patients and 779 healthy controls (Fig. 2). Meta-analysis showed higher levels of WC among POI women compared to the control group (MD = 1.78 [0.74 to 2.83], *P* = 0.0008; I^2^ = 31%). The funnel plot showed no obvious asymmetry, with no evidence of publication bias (Supplementary Fig. 1). The Egger’s line regression test did not indicate publication bias (t = -0.87, *P* = 0.447). Additionally, sensitivity analysis did not identify any single study which altered the effect size.

### Blood pressure

**Fig. 3:**
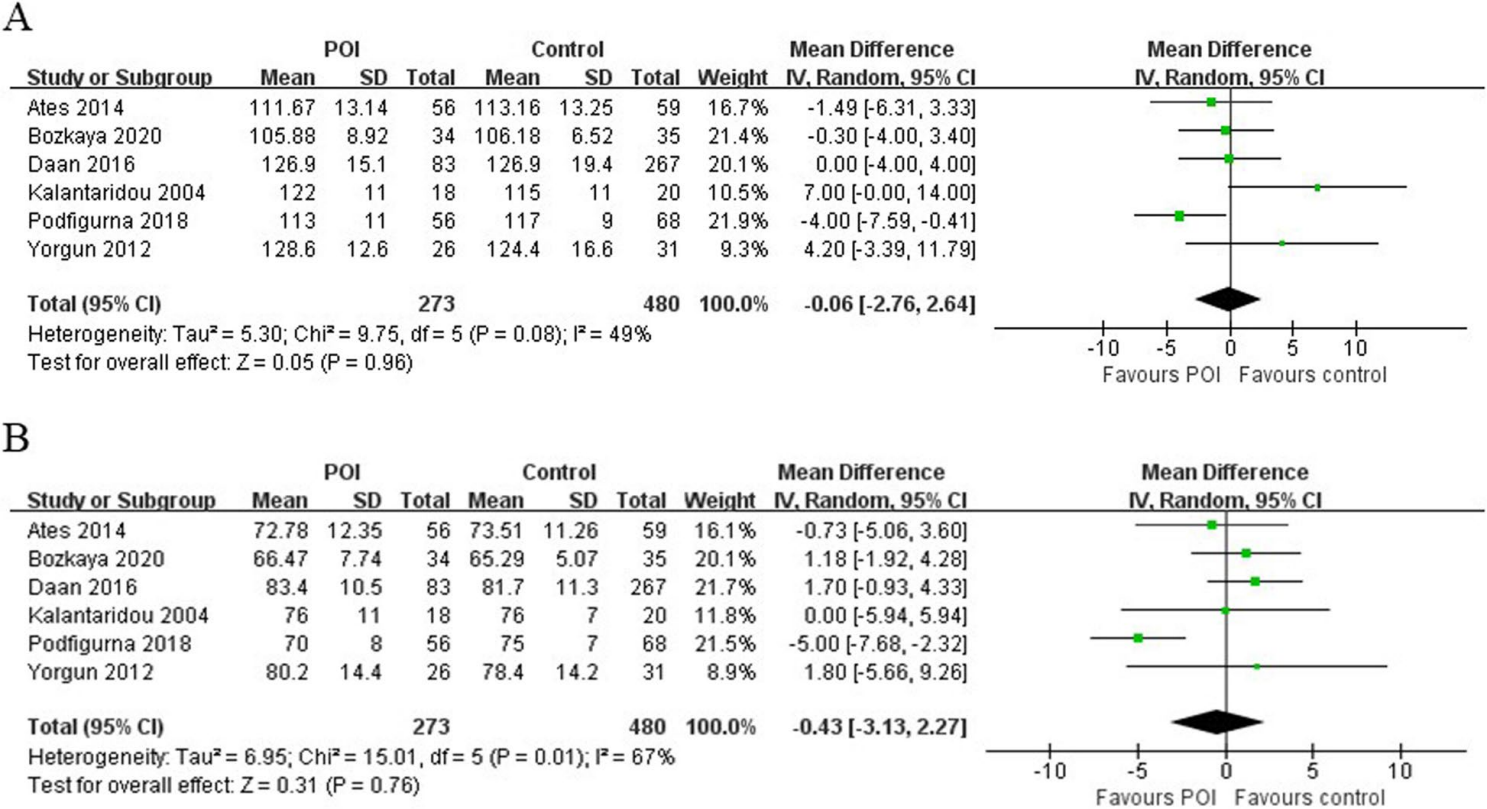
Forest plots for the meta-analysis for (**A**) systolic blood pressure and (**B**) diastolic blood pressure between premature ovarian insufficiency and control group

SBP and DBP were measured in 6 of the included studies which included 273 POI patients and 480 healthy women. SBP (MD = -0.06 [-2.76 to 2.64], *P* = 0.96; I^2^ = 49%) and DBP (MD = -0.43 [-3.13 to 2.27], *P* = 0.76; I^2^ = 67%) were not statistically different between POI women and control women (Fig. 3). The funnel plots showed no obvious asymmetry, with no evidence of publication bias (Supplementary Figs. 2 and 3). The Egger’s line regression test did not indicate publication bias for SBP and DBP (t = 2.44, *P* = 0.092; t = -0.87, *P* = 0.446). Additionally, sensitivity analysis did not identify any single study which altered the effect size.

### Glucose and insulin

**Fig. 4:**
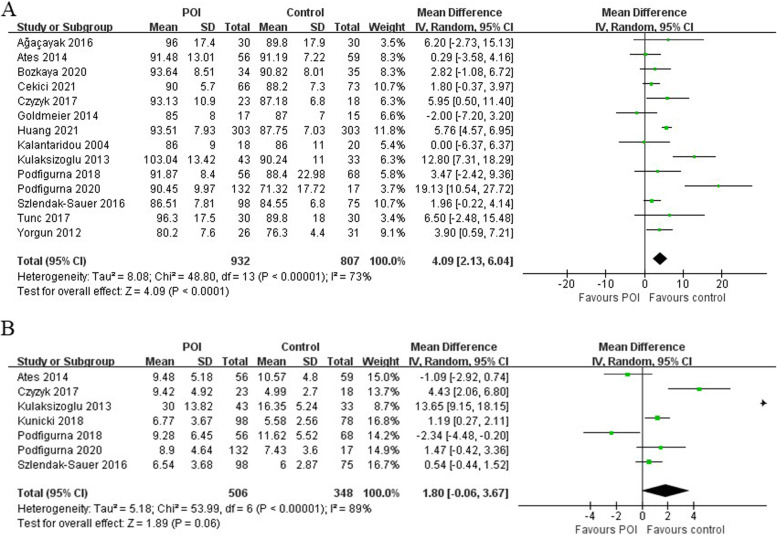
Forest plots for the meta-analysis for (**A**) fasting glucose and (**B**) insulin between premature ovarian insufficiency and control group

Meta-analysis of 14 studies revealed a significantly higher level of FG (MD = 4.09 [2.13 to 6.04], *P* =  < 0.0001; I^2^ = 73%) in patients with POI (*n* = 932) compared to the controls (*n* = 807) (Fig. 4). INS (MD = 1.80 [-0.06 to 3.67], *P* = 0.06; I^2^ = 89%) was measured in 7 of the studies and was marginally higher among patients with POI (*n* = 506) than controls (*n* = 348) (Fig. 4). The funnel plots showed no obvious asymmetry, with no evidence of publication bias (Supplementary Figs. 4 and 5). The Egger’s line regression test did not indicate publication bias for FG and INS (t = 2.44, *P* = 0.092; t = 1.85, *P* = 0.138). Furthermore, sensitivity analysis did not identify any single study which altered the effect size.

### Serum lipid

**Fig. 5:**
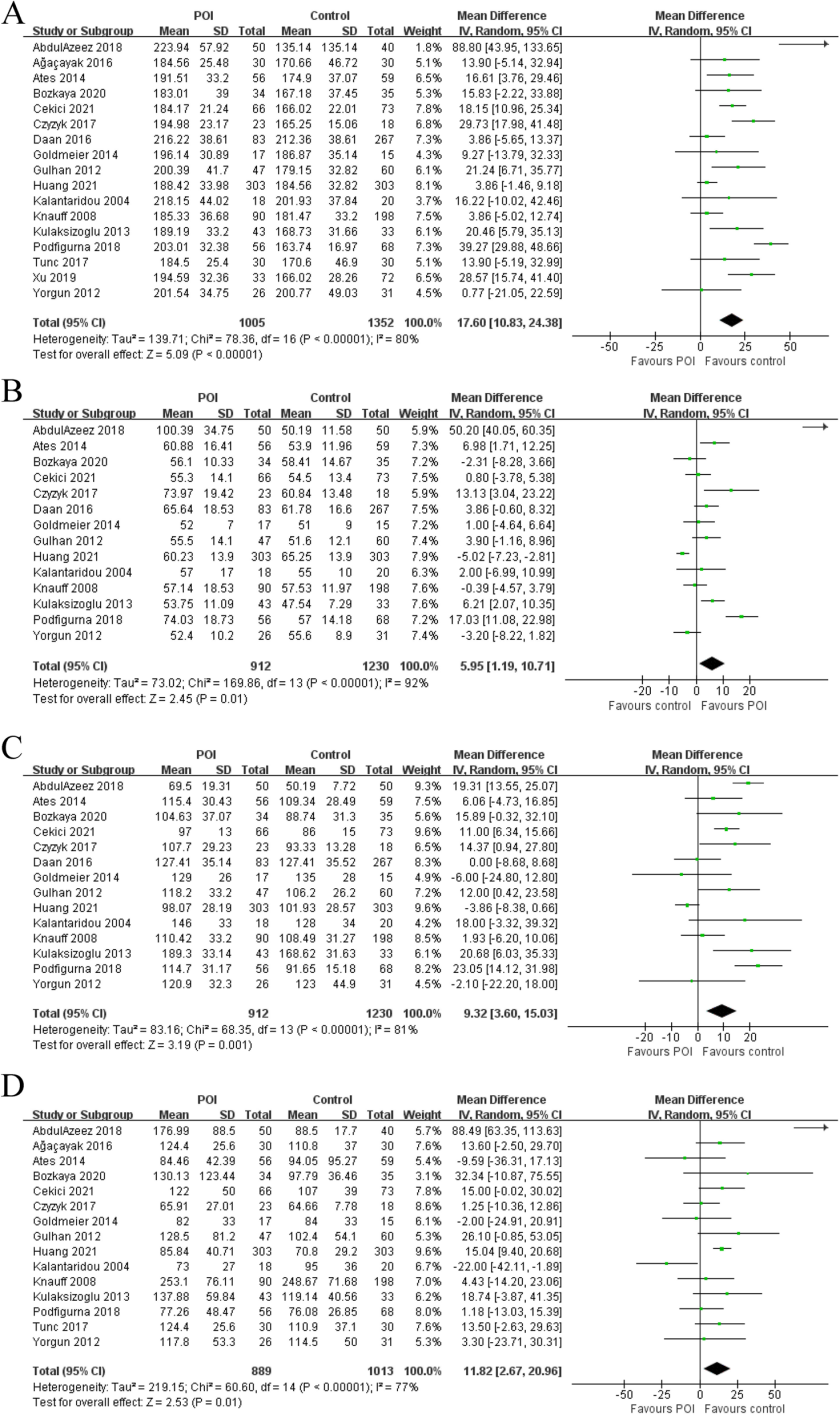
Forest plots for the meta-analysis for (**A**) total cholesterol, (**B**) high-density lipoprotein, (**C**) low-density lipoprotein and (**D**) triglycerides between premature ovarian insufficiency and control group

Meta-analysis of 17 studies revealed a significantly higher level of TC (MD = 17.60 [10.83 to 24.38], *P* =  < 0.00001; I^2^ = 80%) in patients with POI (*n* = 1005) compared to the controls (*n* = 1352) (Fig. 5). Meta-analysis of 14 studies revealed a significantly higher level of HDL (MD = 5.95 [1.19 to 10.71], *P* = 0.01; I^2^ = 92%) in patients with POI (*n* = 912) compared to the controls (*n* = 1230) (Fig. 5). Meta-analysis of 14 studies revealed a significantly higher level of LDL (MD = 9.32 [3.60 to 15.03], *P* = 0.001; I^2^ = 81%) in patients with POI (*n* = 912) compared to the controls (*n* = 1230) (Fig. 5). Meta-analysis of 15 studies revealed a significantly higher level of TG (MD = 11.82 [2.67 to 20.96], *P* = 0.01; I^2^ = 77%) in patients with POI (*n* = 889) compared to the controls (*n* = 1013) (Fig. 5). The funnel plots showed no obvious asymmetry, with no evidence of publication bias, except for HDL (Supplementary Figs. 6, 7, 8 and 9). The Egger’s line regression test did not indicate publication bias for TC, LDL and TG (t = 1.58, *P* = 0.134; t = 0.64, *P* = 0.537; t = 0.01, *P* = 0.991); however, a potential publication bias for HDL (t = 3.48, *P* = 0.005) was noted. Sensitivity analysis did not identify any single study which altered the effect size.

### Subgroup analysis and meta-regression

Subgroup analyses were performed in studies with POI women of normal mean BMI (20 =  < BMI < 25). Meta-analysis of 2 studies revealed WC in patients with POI (*n* = 293) were not different compared to the controls (*n* = 438) (MD = 0.83 [-2.17 to 3.82], *P* = 0.59; I^2^ = 68%). Meta-analysis of 7 studies revealed a significantly higher level of TC in patients with POI (*n* = 586) compared to the controls (*n* = 710) (MD = 17.81 [6.59 to 29.04], *P* = 0.002; I^2^ = 89%). Meta-analysis of 6 studies revealed a significantly altered level of LDL in patients with POI (*n* = 556) compared to the controls (*n* = 680) (MD = 9.65 [0.64 to 18.65], *P* = 0.04; I^2^ = 88%). Meta-analysis showed HDL (MD = 4.06 [-2.63 to 10.74], *P* = 0.23; I^2^ = 91%) and TG (MD = 5.66 [-2.80 to 14.12], *P* = 0.19; I^2^ = 66%) were not different in women with POI compared to control women. Meta-analysis of 8 studies revealed a significantly altered level of FG in patients with POI compared to the controls (MD = 4.42 [1.91 to 6.93], *P* = 0.0005; I^2^ = 76%). INS was measured in 5 of the studies and was not different among patients with POI than controls (MD = 0.99 [-0.43 to 2.41], *P* = 0.17; I^2^ = 79%). These suggested that metabolic differences were significant independent of overweight or obesity.

Univariate meta-regression suggested that estradiol level was associated with the effect size of HDL (coefficient: 0.42 (0.20 to 0.65), *P* = 0.001) and TG (coefficient: 0.60 (0.09 to 1.12), *P* = 0.026), and FSH was associated with the effect size of TG (coefficient: -0.68 (-1.23 to -0.12), *P* = 0.021) (Supplementary Table 3). These suggested that hormone levels had significant impact on the effect size.

## Discussion

Together, the meta-analysis data described in this review highlights the unfavorable metabolic profile observed in POI patients relative to healthy control women including higher WC, FG, TC, LDL, and TG. Since these parameters are closely related to the long-term CVD risk, it is necessary to have early screening and management of metabolic health of women with POI.

First explanation of the association between metabolic abnormalities and POI was sex hormone. In agreement with this explanation, our meta-regression results indicated that hormone levels are associated with the metabolic parameters. Hormonal changes during the menopause transition may facilitate an unfavorable metabolic profile that is characterized by increased TC, LDL, TG, and decreased HDL [35]. Estrogen tends to have a protective role in insulin resistance and metabolic homeostasis [36, 37]. Prolonged estrogen deprivation is associated with an increased estimated risk of CVD in women with POI [38]. Estrogen deficiency promotes metabolic dysfunction predisposing patients to obesity, metabolic syndrome, and type 2 diabetes [37]. Mouse studies also demonstrated that ovariectomy impairs hepatic glucose and lipid metabolism and alters the gut microbiota [39]. Additionally, FSH and its receptors have been reported to have an association with metabolic health [40] and have been implicated in the induction of metabolic diseases through multiple pathways including adipose accumulation, and contribute to obesity, diabetes, and non-alcoholic fatty liver disease [41]. Together, POI may result in a similarly altered metabolic profile compared to women with normal ovarian function due to estrogen deficiency. Therefore, these results support the clinical strategy of prescribing additional hormone therapy to improve metabolic parameters in women with POI and improve health outcomes. However, analysis between POI women using and not using hormone therapy was not available because most included studies didn’t mention whether the women used hormone therapy. Future studies should focus on the effect of hormone therapy on metabolic parameters of women with POI.

Second, steroidogenesis may also contribute to the metabolic abnormalities observed in women with POI. Steroidogenesis is a process in which ovary produces estrogen from cholesterol. The oocyte relies on serum lipids from the maternal circulation to provide cholesteryl esters for granulosa cell steroidogenesis. Failure to produce estrogen at a physiological level may result in the accumulation of substrate lipids in circulation. Interestingly, we found that women with POI had significantly higher levels of HDL compared to control women, possibly because that HDL is the main transporter of cholesterol to granulosa cells during steroidogenesis [42]. We hypothesize that women with POI may have more lipid accumulation in circulation due to the decreased synthesis of estrogen.

There were also other confounders that might influence our results. First, not all confounders were fully adjusted in included studies. Previous studies reported that age and BMI are strongly associated with lipid level [43, 44]. However, age and BMI were not all statistically insignificant between POI and control women among included studies. Other confounders that may affect a person’s metabolic profile, such as smoking [45], hormone therapy [46], lifestyle [47] were not evaluated in most studies. Future studies should focus on the effects of these possible confounders on metabolic parameters in women with POI.

Besides gynecological symptoms of estrogen deficiency, POI is also associated with CVD risk. Obesity is a major risk factor for cardiometabolic abnormalities. The mechanism between obesity and cardiovascular health might be explained by processes including chronic inflammation, insulin resistance, endothelial dysfunction, coronary calcification and so on [48]. The results of our study suggested that metabolic parameters including WC, FG, TC, LDL and TG were altered in women with POI independent of overweight and obesity, which are all risk factors for CVD [49–54]. Although exogenous hormone therapies including contraception pill and hormone replacement therapy are usually associated with cardiovascular events [55], they have potential benefit on metabolic parameters for women with POI [56].

In naturally postmenopausal women, hormone replacement therapy has been hypothesized to have long-term benefits on cardiovascular health [57]. Hormone replacement therapy might improve endothelial dysfunction [24], and reduce blood pressure, plasma angiotensin, and serum creatinine in women with POI [58]. However, in young women with POI undergoing hormone replacement therapy, no long-term data are available to substantiate cardiovascular outcomes. In the current review, we show that even young and lean women with POI were associated with an altered metabolic profile. The screening and prevention of metabolic abnormalities may provide health benefits for women with POI. However, more prospective research is needed to assess if interventions to treat hyperglycemia, dyslipidemia, insulin resistance can bring long-term benefits on cardiovascular for women with POI.

The infertility of POI patients is mainly caused by the reduced quantity and quality of oocytes. Additionally, the chance for spontaneous pregnancy is estimated in 4–10% of women with POI [59]. Currently, there is no treatment for infertility in women with POI. Previous studies have suggested that metabolic abnormalities are associated with female reproductive health and that altered lipids may impair endometrial receptivity [60]. Furthermore, dyslipidemia and metabolic syndrome were associated with a lower live birth rate in infertile women undergoing assisted reproduction [61, 62]. These lines of evidence suggest that interventions designed for metabolic parameters might bring reproductive benefits for women with POI who seek infertility treatment.

Our study has several limitations. The sample size on some indices was relatively small. Most studies were case–control studies and we are unable to fully access the causality between metabolic parameters and POI. Additionally, the quality of included studies were not very high. Lastly, some covariates that may affect a person’s metabolic profile, such as smoking, hormone therapy, lifestyle, were not evaluated in most studies.

## Conclusion

In conclusion, women with POI exhibited increased waist circumference, higher serum lipids, and increased glucose levels. Our study provides improved insight into the understanding of the pathophysiology in women with POI. Future studies are warranted to further explore the underlying mechanism between metabolic abnormalities and POI.

## Supplementary Information

**Additional file 1: Supplementary Figure 1.** Funnel plot in themeta-analysis on the association of waist circumference between prematureovarian insufficiency and control group. **Supplementary Figure 2. **Funnel plot in the meta-analysis on the association of systolic blood pressure betweenpremature ovarian insufficiency and control group. **Supplementary Figure 3. **Funnel plot in the meta-analysis on the association of diastolic blood pressurebetween premature ovarian insufficiency and control group. **Supplementary Figure 4. **Funnel plot in the meta-analysis on the association of fasting glucose betweenpremature ovarian insufficiency and control group. **Supplementary Figure 5. **Funnel plot in the meta-analysis on the association of insulin betweenpremature ovarian insufficiency and control group. **Supplementary Figure 6.**Funnel plot in the meta-analysis on the association of total cholesterol betweenpremature ovarian insufficiency and control group. **Supplementary Figure 7. **Funnel plot in the meta-analysis on the association of high-density lipoproteinbetween premature ovarian insufficiency and control group. **Supplementary Figure 8. **Funnel plot in the meta-analysis on the association of low-density lipoprotein betweenpremature ovarian insufficiency and control group. **Supplementary Figure 9. **Funnel plot in the meta-analysis on the association of triglycerides betweenpremature ovarian insufficiency and control group.

**Additional file 2: Supplementary table 1. **PRISMA 2022 checklist.

**Additional file 3: Supplementary table 2. **Search strategy.

**Additional file 4: Supplementary table 3. **Meta-regression results.

## Data Availability

The datasets used and/or analysed during the current study are available from the corresponding author on reasonable request.

## Abbreviations

POI: Premature ovarian insufficiency
CVD: Cardiovascular disease
PRISMA: Preferred Reporting Items for Systematic Reviews and Meta-analyses
WC: Waist circumference
SBP: Systolic blood pressure
DBP: Diastolic blood pressure
FG: Fasting glucose
INS: Insulin
TC: Total cholesterol
HDL: High-density lipoprotein
LDL: Low-density lipoprotein
TG: Triglycerides
BMI: Body mass index
FSH: Follicle-stimulating hormone
E2: Estradiol
NOS: Newcastle–Ottawa scale
MD: Mean difference
CI: Confidence interval
