# Supplementary material for: Metabolic differences in women with premature ovarian insufficiency: a systematic review and meta-analysis
Source: J Ovarian Res. 2022 Sep 30;15:109. doi: 10.1186/s13048-022-01041-w (PMC9524128; doi:10.1186/s13048-022-01041-w)
Supplement: Supplementary file 1 — Additional file 1: Supplementary Figure 1. Funnel plot in themeta-analysis on the association of waist circumference between prematureovarian insufficiency and control group. Supplementary Figure 2. Funnel plot in the meta-analysis on the association of systolic blood pressure betweenpremature ovarian insufficiency and control group. Supplementary Figure 3. Funnel plot in the meta-analysis on the association of diastolic blood pressurebetween premature ovarian insufficiency and control group. Supplementary Figure 4. Funnel plot in the meta-analysis on the association of fasting glucose betweenpremature ovarian insufficiency and control group. Supplementary Figure 5. Funnel plot in the meta-analysis on the association of insulin betweenpremature ovarian insufficiency and control group. Supplementary Figure 6.Funnel plot in the meta-analysis on the association of total cholesterol betweenpremature ovarian insufficiency and control group. Supplementary Figure 7. Funnel plot in the meta-analysis on the association of high-density lipoproteinbetween premature ovarian insufficiency and control group. Supplementary Figure 8. Funnel plot in the meta-analysis on the association of low-density lipoprotein betweenpremature ovarian insufficiency and control group. Supplementary Figure 9. Funnel plot in the meta-analysis on the association of triglycerides betweenpremature ovarian insufficiency and control group. [file 13048_2022_1041_MOESM1_ESM.docx]

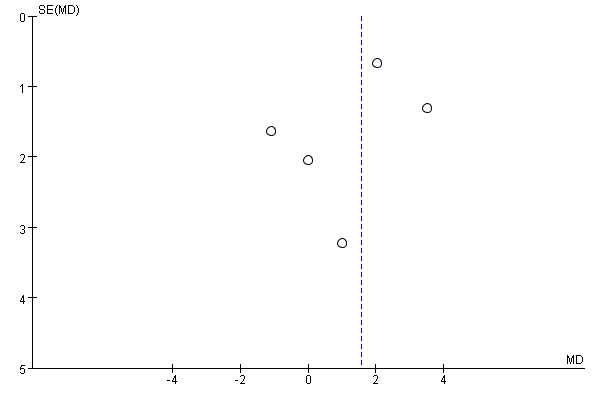


**Supplementary Figure 1** Funnel plot in the meta-analysis on the association of waist circumference between premature ovarian insufficiency and control group
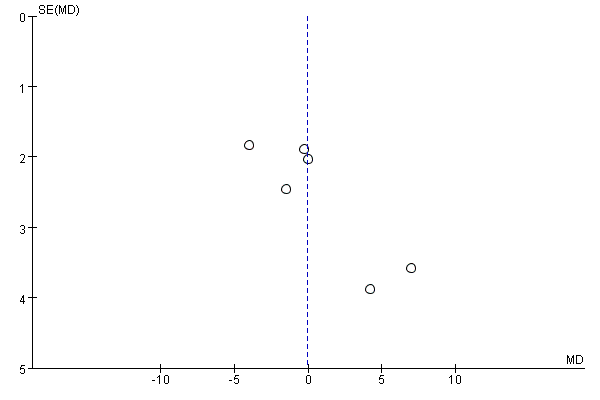


**Supplementary Figure 2** Funnel plot in the meta-analysis on the association of systolic blood pressure between premature ovarian insufficiency and control group


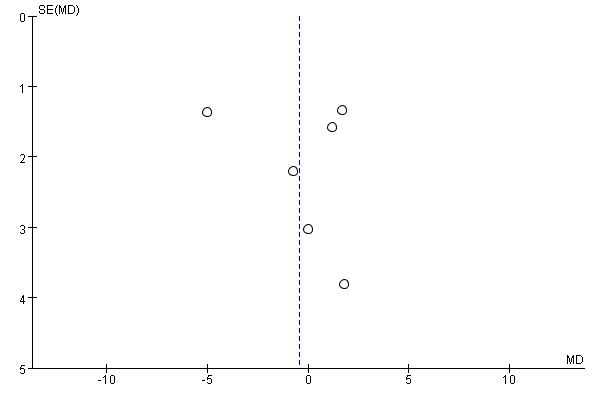


**Supplementary Figure 3** Funnel plot in the meta-analysis on the association of diastolic blood pressure between premature ovarian insufficiency and control group


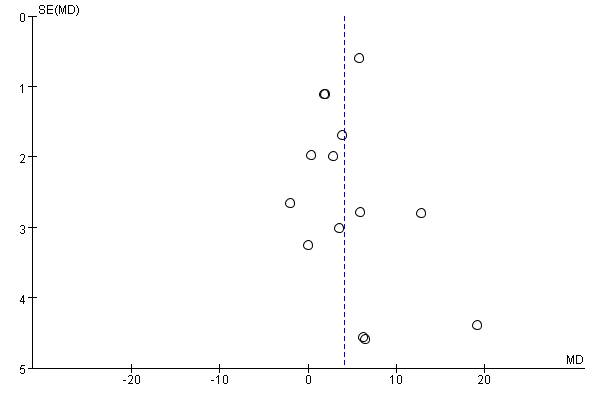


**Supplementary Figure 4** Funnel plot in the meta-analysis on the association of fasting glucose between premature ovarian insufficiency and control group


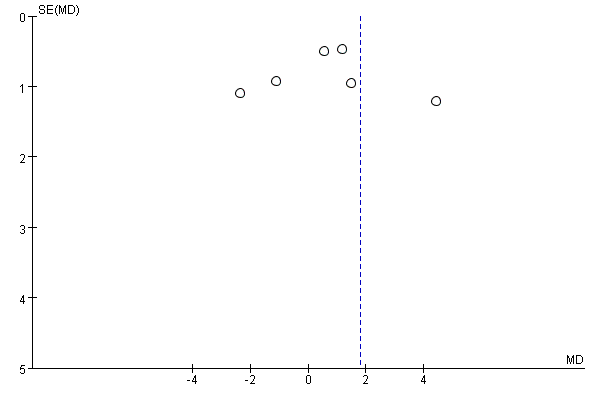


**Supplementary Figure 5** Funnel plot in the meta-analysis on the association of insulin between premature ovarian insufficiency and control group


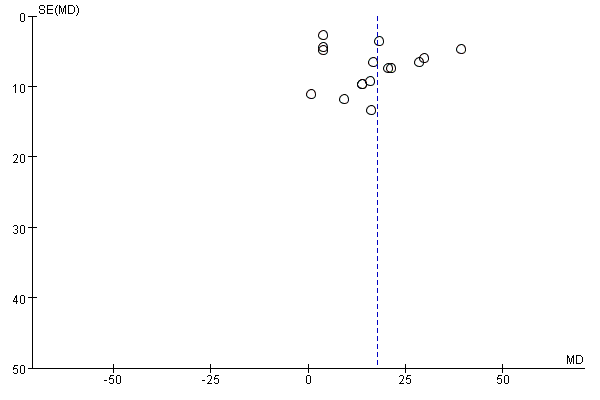


**Supplementary Figure 6** Funnel plot in the meta-analysis on the association of total cholesterol between premature ovarian insufficiency and control group


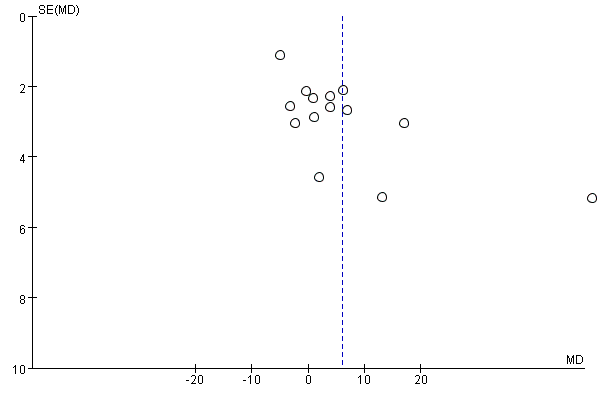


**Supplementary Figure 7** Funnel plot in the meta-analysis on the association of high-density lipoprotein between premature ovarian insufficiency and control group


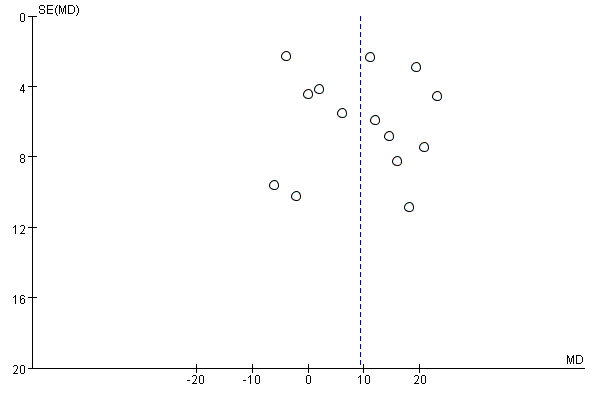


**Supplementary Figure 8** Funnel plot in the meta-analysis on the association of low-density lipoprotein between premature ovarian insufficiency and control group


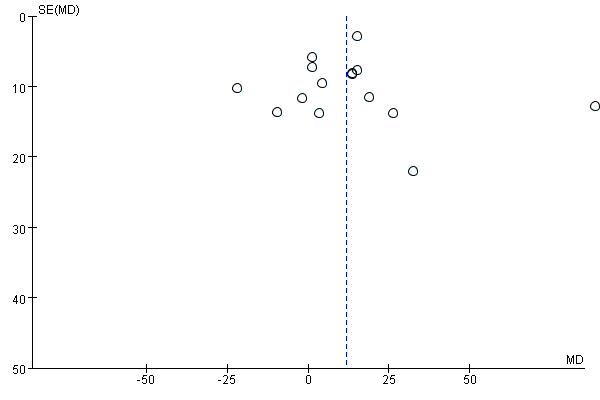


**Supplementary Figure 9** Funnel plot in the meta-analysis on the association of triglycerides between premature ovarian insufficiency and control group
