# Supplementary material for: Metabolic differences in women with premature ovarian insufficiency: a systematic review and meta-analysis
Source: J Ovarian Res. 2022 Sep 30;15:109. doi: 10.1186/s13048-022-01041-w (PMC9524128; doi:10.1186/s13048-022-01041-w)
Supplement: Supplementary file 3 — Additional file 3: Supplementary table 2. Search strategy. [file 13048_2022_1041_MOESM3_ESM.docx]

Pubmed

| #1 | (Primary Ovarian Insufficiency[Mesh]) OR (Menopause, Premature[Mesh]) OR (menopause[Mesh]) OR (premature ovarian insufficiency[Title/Abstract]) OR (premature ovarian failure[Title/Abstract]) OR (diminished ovarian reserve[Title/Abstract]) OR (poor ovarian response[Title/Abstract]) OR (hyper-gonadotropic hypogonadism[Title/Abstract]) OR (elevated gonadotrophins[Title/Abstract]) OR (triad of amenorrhea[Title/Abstract]) OR (estrogen deficiency[Title/Abstract]) |
| --- | --- |
| #2 | (metabolic syndrome[Mesh]) OR (blood pressure[Mesh]) OR (hypertension[Mesh]) OR (glucose[Mesh]) OR (hyperglycemia[Mesh]) OR (insulin[Mesh]) OR (diabetes mellitus[Mesh]) OR (waist circumference[Mesh]) OR (Lipids[Mesh]) OR (Hyperlipidemias[Mesh]) OR (cholesterol[Mesh]) OR (triglycerides[Mesh]) OR (cholesterol, LDL[Mesh]) OR (cholesterol, HDL[Mesh]) OR (metabolic[Title/Abstract]) OR (blood pressure[Title/Abstract]) OR (hypertension[Title/Abstract]) OR (glucose[Title/Abstract]) OR (insulin[Title/Abstract]) OR (hyperglycemia[Title/Abstract]) OR (diabetes mellitus[Title/Abstract]) OR (waist circumference[Title/Abstract]) OR (Lipid[Title/Abstract]) OR (Hyperlipidemias[Title/Abstract]) OR (cholesterol[Title/Abstract]) OR (triglycerides[Title/Abstract]) OR (LDL[Title/Abstract]) OR (HDL[Title/Abstract]) |
| #3 | (Case-Control Studies[Mesh]) OR (Cohort Studies[Mesh]) OR (control[Title/Abstract]) OR (cohort[Title/Abstract]) |
| #4 | #1 AND #2 AND #3 |

Embase

| #1 | ' Menopause, Premature '/exp OR ' Primary Ovarian Insufficiency '/exp OR 'premature ovarian insufficiency ':ab,ti OR ' premature ovarian failure ':ab,ti OR ' diminished ovarian reserve ':ab,ti OR ' poor ovarian response ':ab,ti OR ' hyper-gonadotropic hypogonadism ':ab,ti OR 'reproductive techniques':ab,ti OR 'art':ab,ti OR 'blastocyst transfer':ab,ti OR ' elevated gonadotrophins ':ab,ti OR ' triad of amenorrhea ':ab,ti OR ' estrogen deficiency ':ab,ti |
| --- | --- |
| #2 | 'metabolic syndrome X'/exp OR 'blood pressure'/exp OR 'hypertension'/exp OR 'glucose'/exp OR 'hyperglycemia'/exp OR 'insulin'/exp OR 'diabetes mellitus'/exp OR 'waist circumference'/exp OR 'lipid'/exp OR 'cholesterol'/exp OR 'triacylglycerol'/exp OR 'low density lipoprotein'/exp OR 'high density lipoprotein'/exp OR 'metabolic':ab,ti OR 'blood pressure':ab,ti OR 'hypertension':ab,ti OR 'glucose':ab,ti OR 'hyperglycemia':ab,ti OR 'diabetes mellitus':ab,ti OR 'waist circumference':ab,ti OR 'Lipids':ab,ti OR 'Hyperlipidemias':ab,ti OR 'cholesterol':ab,ti OR 'triglycerides':ab,ti OR 'LDL':ab,ti OR 'HDL':ab,ti |
| #3 | 'case control study'/exp OR 'cohort analysis'/exp OR 'control':ab,ti OR 'cohort':ab,ti |
| #4 | #1 AND #2 AND #3 |

WOS

| #1 | Topic: (Menopause, Premature) OR Topic:(Primary Ovarian Insufficiency) OR Topic:(premature ovarian insufficiency) OR Topic: (premature ovarian failure) OR Topic:(diminished ovarian reserve) OR Topic: (poor ovarian response) OR Topic:( hyper-gonadotropic hypogonadism) OR Topic: (elevated gonadotrophins) OR Topic: (triad of amenorrhea) OR Topic:(estrogen deficiency) |
| --- | --- |
| #2 | Topic: (metabolic syndrome) OR Topic: (blood pressure) OR Topic: (hypertension) OR Topic: (glucose) OR Topic: (hyperglycemia) OR Topic: (insulin) OR Topic: (diabetes mellitus) OR Topic: (waist circumference) OR Topic: (Lipids) OR Topic: (Hyperlipidemias) OR Topic: (cholesterol) OR Topic: (triglycerides) OR Topic: (LDL) OR (HDL) |
| #3 | Topic: (Control) OR Topic: (Cohort) |
| #4 | #1 AND #2 AND #3 |
