# Supplementary material for: Metabolic differences in women with premature ovarian insufficiency: a systematic review and meta-analysis
Source: J Ovarian Res. 2022 Sep 30;15:109. doi: 10.1186/s13048-022-01041-w (PMC9524128; doi:10.1186/s13048-022-01041-w)
Supplement: Supplementary file 4 — Additional file 4: Supplementary table 3. Meta-regression results. [file 13048_2022_1041_MOESM4_ESM.docx]

| Supplementary table 3 Meta-regression results | | | | | | |
| --- | --- | --- | --- | --- | --- | --- |
| Outcomes | Moderator | Number of studies | Number of POI | Number of controls | Coefficient (95% confidence interval) | P value |
| WC | Age | 5 | 449 | 779 | 0.13 (-0.28 to 0.55) | 0.379 |
|  | E2 | 5 | 449 | 779 | -0.11 (-0.26 to 0.05) | 0.114 |
|  | FSH | 4 | 366 | 512 | 0.08 (-0.24 to 0.40) | 0.400 |
| SBP | Age | 5 | 217 | 412 | -0.03 (-0.75 to 0.70) | 0.915 |
|  | E2 | 5 | 217 | 412 | 0.00 (-0.34 to 0.34) | 0.998 |
|  | FSH | 4 | 134 | 145 | 0.02 (-0.81 to 0.86) | 0.919 |
| DBP | Age | 5 | 217 | 412 | 0.06 (-0.28 to 0.38) | 0.628 |
|  | E2 | 5 | 217 | 412 | -0.05 (-0.21 to 0.12) | 0.450 |
|  | FSH | 4 | 217 | 412 | -0.08 (-0.53 to 0.38) | 0.532 |
| FG | Age | 13 | 876 | 739 | -0.25 (-1.32 to 0.83) | 0.623 |
|  | E2 | 13 | 876 | 739 | -0.12 (-0.36 to 0.12) | 0.302 |
|  | FSH | 13 | 876 | 739 | 0.06 (-0.15 to 0.28) | 0.520 |
| INS | Age | 6 | 450 | 280 | 1.17 (-0.73 to 3.07) | 0.175 |
|  | E2 | 6 | 450 | 280 | -0.15 (-0.59 to 0.28) | 0.406 |
|  | FSH | 6 | 450 | 280 | -0.16 (-0.42 to 0.09) | 0.159 |
| TC | Age | 16 | 972 | 1280 | -1.24 (-2.56 to 0.06) | 0.060 |
|  | E2 | 16 | 972 | 1280 | 0.43 (-0.00 to 0.86) | 0.051 |
|  | FSH | 16 | 922 | 1085 | 0.06 (-0.40 to 0.53) | 0.766 |
| LDL | Age | 14 | 912 | 1230 | -0.92 (-1.83 to 0.00) | 0.051 |
|  | E2 | 14 | 912 | 1230 | 0.13 (-0.07 to 0.34) | 0.179 |
|  | FSH | 13 | 829 | 963 | -0.01 (-0.30 to 0.28) | 0.940 |
| HDL | Age | 14 | 912 | 1230 | -1.15 (-2.53 to 0.23) | 0.095 |
|  | E2 | 14 | 912 | 1230 | 0.42 (0.20 to 0.65) | **0.001** |
|  | FSH | 13 | 829 | 963 | -0.22 (-0.62 to 0.18) | 0.255 |
| TG | Age | 15 | 889 | 1013 | -3.41 (-6.91 to 0.08) | 0.055 |
|  | E2 | 15 | 889 | 1013 | 0.60 (0.09 to 1.12) | **0.026** |
|  | FSH | 15 | 889 | 1013 | -0.68 (-1.23 to -0.12) | **0.021** |
| waist circumference (WC), fasting blood glucose (FG), insulin (INS), systolic blood pressure (SBP), diastolic blood pressure (DBP), total cholesterol (TC), high-density lipoprotein (HDL), low-density lipoprotein (LDL) and triglycerides (TG), FSH (follicle-stimulating hormone), E2 (estradiol), | | | | | | |
